# A Case Report of Losartan Induced Angioedema

**DOI:** 10.7759/cureus.36525

**Published:** 2023-03-22

**Authors:** Venkata Vedantam, Hezborn M Magacha, Neethu Vedantam, Vanessa Dahya, Usama Abu-Heija

**Affiliations:** 1 Internal Medicine, East Tennessee State University - Quillen College of Medicine, Johnson City, USA; 2 General Practice, East Tennessee State University, Johnson City, USA; 3 Infectious Diseases, East Tennessee State University - Quillen College of Medicine, Johnson City, USA

**Keywords:** arb, acei, angiotensin, losartan, angioedema

## Abstract

Angiotensin-converting-enzyme inhibitors (ACEI) and Angiotensin Receptor Blockers (ARBs) are commonly used to manage hypertension and cardiovascular diseases. Although angioedema due to ACEI is a well-known side effect, only a few cases are associated with ARBs, such as losartan. Medication-induced angioedema has been known for many years; however, the mechanism by which many medications cause angioedema is not clearly understood. Here we present the case of angioedema in a 50-year-old male taking losartan after he developed acute kidney disease.

## Introduction

Angiotensin-converting-enzyme inhibitors (ACEI) and Angiotensin Receptor Blockers (ARBs) are commonly used medications for treating pathologies like renal failure, heart failure, and hypertension [1]. One of the side effects caused by the inhibition of Angiotensin-converting-enzymes (ACE) is angioedema, a swelling of the submucosa and subcutaneous layers of tissues of the face, lips, oral cavity, larynx, neck, extremities, and gut [2]. Angioedema is a rare side effect associated with the use of losartan. ARBs act at the angiotensin receptors and have no systemic or local effect on the bradykinin level. Its occurrence with ARBs questions the pathophysiology of angioedema and the safety of this class of drugs as a substitute for those who developed angioedema on ACEI [3].

Here we report a clinical presentation, management, and outcome of a 60-year-old male patient who presented with losartan-induced angioedema.

## Case presentation

A 60-year-old male with a history of resistant hypertension (HTN) on losartan (50mg), clonidine, chlorthalidone, and amlodipine presented to the Emergency Department (ED) with a one-day history of worsening swelling of his face, lips, and tongue. In the ED, the patient was found to have stridor, progressively increasing difficulty in breathing, and blood pressure of 188/115mmHg. On physical examination, the face, tongue, and lips were swelling. There was no urticaria on his body or wheezing on auscultation.

 The patient was given diphenhydramine-50mg, Solu-Medrol 125mg, and intramuscular epinephrine -1mg without improvement in his symptoms and was intubated for airway protection. The only significant laboratory abnormality was a creatinine of 2.8 mg/dL and blood urea nitrogen (BUN) of 42 mg/dL on admission. Laboratory findings as shown in the table below (table 1).

**Table 1:** Laboratory findings MCV: mean corpuscular volume; BUN: blood urea nitrogen

Laboratory values	Patient Values	Reference Values
white blood cell count	10	(3.5-10.5) K/µL
Hemoglobin	9.9	12-16 g/dL
Platelet	262	(150-450) K/µL
MCV	89	80-100µm^3^
Neutrophils	9.5	(1.5-7.5) K/µL
Monocytes	0.67	0-0.8*10^3^/µL
Eosinophils	0.0	0-0.5*10^3^/ µL
Creatinine	2.8	(0.6-1.10) mg/dL
BUN	42	(6-20) mg/dL
Potassium	5.3	(3.5-5.1) mmol/L

All oral blood pressure medications were held, including losartan, chlorthalidone, and clonidine. Two days later, the patient was successfully extubated. The patient’s facial, tongue, and lip swelling had improved significantly. He had allergies to sulfa antibiotics but denied any current use. Upon further inquiry, the patient stated that two months ago, he was diagnosed with rapidly progressive glomerulonephritis (RPGN), for which he was started on 10mg of prednisone by his nephrologist. The patient denied any recent medication changes except that his hydrochlorothiazide was switched to chlorthalidone because of his kidney disease. He stated that he had not taken any ACEI and denied any recent increase in losartan dose or any previous personal/family history of similar events. At discharge, the patient’s facial and tongue swelling resolved entirely. The probability of losartan causing angioedema in this patient was assessed using the Naranjo Adverse Drug Reaction Probability Scale, which eliminated the possibility of all other etiologies for this reaction and correlated the onset with the suspected medication use. The patient was diagnosed with losartan-induced angioedema, and losartan was discontinued at discharge. The patient was educated not to take any ACEI or ARBs.

## Discussion

The ACEIs block the degradation pathway of bradykinins, leading to the accumulation of bradykinins, resulting in increased vascular permeability and swelling of the mucosa, which can persist for a few hours to days [3].

Angioedema is a life-threatening condition that can lead to respiratory compromise secondary to mucosal swelling and requires immediate action [4]. Angioedema can be both hereditary and acquired. The hereditary form is due to the defect in the genes encoding the C1-esterase inhibitor. In contrast, the acquired form can be caused by drugs, infections, neoplasia, and autoimmune or lymphoproliferative disorders [1,5]. ACEI is the most common cause of drug-induced angioedema accounting for 25-50% of all cases. Other medications like ARBs, non-steroidal anti-inflammatory drugs (NSAIDs), aspirin, and certain antibiotics account for a small percentage of drug-induced angioedema. Although drug-induced angioedema has been known for many years, the exact mechanism by which most drugs, other than ACEI, cause angioedema has yet to be clearly explained or extensively studied [[1,6]. Losartan rarely causes angioedema, and when losartan was first approved for use, angioedema was a negligible side effect. Still, with continued use over the years, several cases of patients presenting with this rare side effect have been documented. However, the prevalence remains unknown [1].

 Losartan and other ARBs, unlike ACEI, do not impact the degradation pathway of these cytokines. Some studies postulate that Angiotensin-receptor blocker activates the prostaglandin-bradykinin and nitric-oxide cascade, inducing bradykinin-mediated effects by losartan, such as angioedema, but the whole mechanism by which losartan causes angioedema is not well understood [5]. Animal studies reveal an increased risk of angioedema with losartan use compared to placebo; however, the symptoms are less severe and occur earlier than ACEI-induced angioedema [6,7]. The average time-to-onset of angioedema after losartan use range from 10 hours to 16 years of losartan use. Unlike histamine-mediated allergic reactions, bradykinin-mediated reactions manifest with significant mucosal edema without urticaria-most of the patients present with swelling of the face, lips, and tongue. In severe cases, mucosal edema of the gut will result in abdominal pain, nausea, vomiting, diarrhea, or constipation [8]. The involvement of laryngeal mucosa will result in airway obstruction and respiratory failure. The clinical manifestation, as in the case of our patient, tends to manifest over a few hours to days and can progress rapidly to complications, including airway compromise and death [9]. Prompt discontinuation of the offending medication early in the course, as soon as the diagnosis is suspected, remains the mainstay of treatment. Although antihistamines, H2 blockers, and corticosteroids are the mainstay of managing histamine-mediated allergic reactions, their utility in bradykinin-mediated angioedema remains poor. However, since 30% of histamine-mediated allergic reactions tend to manifest with angioedema alone without urticaria, a trial of these medications can mitigate the need for intubation if the angioedema is histamine mediated. In certain circumstances, as in the case reported here, the medications do not work, and a patient may present with respiratory distress, stridor, tongue edema, drooling, and swelling of the floor of the mouth; in such situations, the patient has to be intubated [10].

Different factors have been associated with losartan-induced angioedema. Studies found that one factor that increases the risk for angioedema in losartan use is the previous use of ACEI medication in patients, a mechanism known as cross-reactivity [2]. In our case, the patient denied using any ACEI medication; hence cross-reactivity was not a factor for this patient. Scientific research reveals that 32% of patients who presented with ARB-induced angioedema had experienced a similar reaction while on ACEI [2,11,12]. According to studies, other factors contributing to angioedema include renal failure [12].

Furthermore, age and drug interactions with medications such as non-steroidal anti-inflammatory drugs are independent risk factors for angioedema [7]. Several studies have identified increasing the dose of losartan for patients who have been taking this medication for many years as a risk factor for angioedema [11]. The risk of medication-induced angioedema is modified by race/ethnicity and sex, whereby the risk is high in African Americans than in other ethnic groups and higher in females compared to males [12].

## Conclusions

Losartan-induced angioedema is a rare side effect; however, because of the widespread use of this medication, it is vital for physicians to be aware of this rare phenomenon and to stop the medication in patients who develop angioedema. This case report shows that losartan and other angiotensin-receptor blockers should be used cautiously as a substitute for Angiotensin-converting enzyme inhibitors. Patients should be aware of this rare side effect. Close monitoring is required whenever patients present with the symptoms, as this rare phenomenon can progress to respiratory failure and lead to death.
